# AntiTNF-alpha therapy normalizes levels of lipids and adipokines in psoriatic patients in the real-life settings

**DOI:** 10.1038/s41598-021-88552-6

**Published:** 2021-04-29

**Authors:** Irmina Olejniczak-Staruch, Joanna Narbutt, Justyna Ceryn, Małgorzata Skibińska, Igor Bednarski, Anna Woźniacka, Joanna Sieniawska, Marzena Kraska-Gacka, Magdalena Ciążyńska, Janusz Śmigielski, Marcin Noweta, Michał Waszczykowski, Witold Owczarek, Adam Reich, Aleksandra Lesiak

**Affiliations:** 1 Department of Dermatology, Pediatric Dermatology and Dermatological Oncology, Medical University of Lodz, Lodz, Poland; 2 Dermoklinika Centrum Medyczne, Lodz, Poland; 3 Department of Dermatology and Venereology, Medical University of Lodz, Lodz, Poland; 4 Department of Proliferative Diseases, Nicolaus Copernicus Multidisciplinary Centre for Oncology and Traumatology, Lodz, Poland; 5 State Higher Vocational School in Konin, Konin, Poland; 6 Department of Arthroscopy, Minimally Invasive Surgery and Sports, Traumatology Medical University of Lodz, Lodz, Poland; 7 Department of Dermatology, Military Institute of Medicine, Warsaw, Poland; 8 Department of Dermatology, University of Rzeszow, Rzeszow, Poland

**Keywords:** Biomarkers, Diseases, Medical research

## Abstract

Studies have shown that the levels of pro-inflammatory adipokines in patients with psoriasis are higher than in general population. The aim of the study was to investigate the influence of 36-month therapy with TNF-α inhibitors (adalimumab, etanercept, infliximab) on the levels of adipokines (resistin, adiponectin, leptin) and lipids (TG, cholesterol, LDL, HDL) in 37 psoriasis patients and 30 healthy controls. The mean serum concentrations of adiponectin in patients from adalimumab, etanercept and infliximab group were similar to control group (*p* > 0.05, 142.71, 164.32, 129.35 and 174.44 μg/ml respectively). Resistin levels were higher in patients (*p* < 0.05, 4.48, 4.53 and 3.39 ng/ml respectively) than in controls (3.05 ng/ml). Mean leptin concentrations were significantly higher (*p* < 0.05) in the study group than in subjects without psoriasis (428.61, 523.24, 755.27 and 154.10 pg/ml respectively). A significant decrease in the mean resistin concentration was observed under the influence of biological therapy (*p* < 0.05). Decrease in serum leptin level was noted in etanercept and infliximab groups (*p* = 0.001 and *p* = 0.002 respectively). Improvement in all lipidogram parameters was noted in all examined groups (*p* < 0.05). Results may prove that biologic therapy affects the systemic inflammation associated with psoriasis and this effect persists with long-term therapy.

## Introduction

Psoriasis is a chronic, recurrent inflammatory skin disease which prevalence varies from 0.91% (United States) to 8.5% (Norway)^1^. Recent findings indicate that inflammation accompanying psoriasis promotes the development of such comorbidities as atherosclerosis, diabetes mellitus or metabolic syndrome^2–4^.

Nowadays, white fatty tissue is regarded as an endocrine organ, which synthesizes biologically active molecules: adipokines, proinflammatory cytokines, procoagulant factors and free fatty acids adipocytokines^5–7^. Adipocytokines exhibit pleiotropic activities, such as regulation of the appetite, lipid and glucose metabolism, coagulation, immunity, fibrinolysis and angiogenesis. Some of them have pro-inflammatory (e.g. tumor necrosis factor- α—TNF- α, leptin and resistin) whereas other anti-inflammatory properties (e.g. adiponectin)^6^. Serum levels of resistin are higher, while adiponectin lower in psoriasis patients in comparison with healthy controls and correlate with psoriasis severity, therefore, can be used as potential biomarkers for psoriasis development and treatment effectiveness^2^.

Better understanding of the pathogenesis of the disease has enabled the creation of targeted therapies—including biological therapy such as TNF-α inhibitors which were proved to be highly effective and well-tolerated in plaque psoriasis in long-term observation^8,9^.

Numerous studies have shown that the levels of pro-inflammatory lipids and adipokines in patients with psoriasis, especially those with a severe course, are higher than in healthy people. It has been proven that they can be modified by systemic psoriasis treatment. However, most observations on the effects of drugs on lipid metabolism in this dermatosis are short-term and their results are inconsistent.

The aim of the study was to investigate the influence of long-term biologic therapy with TNF-α inhibitors (adalimumab, etanercept, infliximab) on the levels of adipokines (resistin, adiponectin, leptin) in relation to PASI (Psoriasis Activity and Severity Index), DLQI (Dermatology Life Quality Index), BMI (Body Mass Index) and lipid profile (TG—triglyceride , TC—total cholesterol, LDL—low density lipoprotein, HDL—high density lipoprotein) change.

## Results

The PASI scoring of patients before treatment with adalimumab was 15.12 ± 4.01 points, with etanercept—13.9 ± 3.41 points, and infliximab—13.51 ± 3.73 points. After a 3-month follow-up period, patients receiving biological treatment experienced a significant reduction in the severity of skin lesions as measured with PASI in all groups (adalimumab—3.13 ± 2.74 points, etanercept—3.81 ± 3.44 points, and infliximab 5.1 ± 4.97 points). The clinical effect was maintained until the 36th month of therapy with all drugs (PASI: 2.98 ± 1.78 points, 2.52 ± 0.60 points, and 2.84 ± 2.13 points, respectively) (Table 1).

**Table 1:** Parameters in the control group and in psoriasis group under biologic therapy at baseline and in 3., 12., 24., 36. month of therapy.

Parameter	Control	Study group	Baseline	3. month	12. month	24. month	36. month	Control vs baseline	Repeated measures (*p* value)
PASI		Adalimumab	15.12 ± 0.94	3.13 ± 0.63	2.38 ± 0.48	2.72 ± 0.27	2.98 ± 0.41		< 0.0001*
		Etanercept	13.90 ± 3.41	3.81 ± 3.44	3.28 ± 2.03	1.96 ± 1.38	2.52 ± 0.6		0.00014*
		Infliximab	13.51 ± 3.73	5.10 ± 4.97	3.90 ± 2.93	2.51 ± 2.73	2.84 ± 2.13		0.00219*
DLQI		Adalimumab	16.53 ± 2.88	6.16 ± 1.8	3.05 ± 1.31	2.74 ± 1.56	2.00 ± 1.20		< 0.0001*
		Etanercept	17.92 ± 3.55	6.67 ± 2.43	3.25 ± 1.49	2.33 ± 1.56	2.17 ± 1.34		< 0.0001*
		Infliximab	17.83 ± 3.49	7.67 ± 1.63	2.67 ± 1.86	2.50 ± 1.52	1.50 ± 1.38		< 0.0001*
BMI (kg/m^2^)	24.41 ± 0.87	Adalimumab	28.76 ± 0.93	28.75 ± 0.99	28.63 ± 1.01	29.01 ± 0.97	28.96 ± 0.97	0.0022*	0.7408
		Etanercept	28.02 ± 3.05	28.08 ± 3.67	27.73 ± 3.75	28.02 ± 3.57	27.81 ± 3.41	0.0069*	0.48258
		Infliximab	28.11 ± 3.15	28.04 ± 3.81	27.61 ± 3.74	28.29 ± 3.55	27.91 ± 3.09	0.0830	0.11433
TG (mg/dl)	102.83 ± 28.85	Adalimumab	147.42 ± 64.87	126.89 ± 45.99	134.68 ± 56.00	123.11 ± 43.53	120.84 ± 46.12	0.0097*	0.0051*
		Etanercept	164.45 ± 41.06	143.36 ± 37.80	142.55 ± 44.92	136.09 ± 38.53	124.36 ± 38.26	0.0001*	0.00003*
		Infliximab	162.86 ± 39.83	150.29 ± 37.55	151.14 ± 48.68	143.57 ± 38.69	137.86 ± 41.87	0.0044*	0.00306*
TC (mg/dl)	163.67 ± 21.08	Adalimumab	223.74 ± 45.53	205.00 ± 50.12	221.26 ± 51.52	205.05 ± 45.52	202.32 ± 43.56	0.0001*	0.0049*
		Etanercept	227.00 ± 43.75	187.36 ± 40.07	194.09 ± 46.54	180.18 ± 41.37	169.00 ± 47.98	< 0.0001*	< 0.0001*
		Infliximab	212.29 ± 40.08	175.71 ± 36.35	182.57 ± 39.79	169.43 ± 36.13	167.71 ± 33.52	0.0084*	0.00201*
LDL (mg/dl)	68.93 ± 14.87	Adalimumab	119.05 ± 53.98	100.16 ± 42.60	119.11 ± 52.13	106.11 ± 42.98	103.00 ± 39.81	0.0005*	0.0069*
		Etanercept	115.82 ± 33.86	91.55 ± 25.81	96.82 ± 35.67	89.64 ± 24.97	84.36 ± 32.91	< 0.0001*	0.00002*
		Infliximab	105.71 ± 15.34	79.00 ± 12.92	82.29 ± 20.14	75.29 ± 9.33	74.43 ± 11.18	< 0.0001*	0.00259*
HDL (mg/dl)	64.30 ± 12.72	Adalimumab	54.21 ± 13.93	59.58 ± 15.62	53.84 ± 9.71	54.63 ± 8.73	58.05 ± 14.19	0.0164*	0.1076
		Etanercept	47.36 ± 11.08	53.82 ± 13.06	55.55 ± 11.51	57.64 ± 11.0	57.00 ± 12.49	0.0021*	0.00226*
		Infliximab	53.14 ± 6.54	68.71 ± 10.24	64.00 ± 8.41	63.86 ± 7.67	73.71 ± 4.89	0.0451*	0.00174*
Adiponectin (μg/ml)	174.44 ± 23.50	Adalimumab	142.71 ± 21.23	138.19 ± 17.68	125.79 ± 14.57	131.70 ± 18.62	150.83 ± 19.89	0.3215	0.2551
		Etanercept	164.32 ± 107.51	166.78 ± 82.19	201.61 ± 102.58	204.65 ± 101.34	199.85 ± 103.44	> 0.999	0.19645
		Infliximab	129.35 ± 63.73	141.53 ± 61.53	121.68 ± 64.41	139.60 ± 65.45	152.42 ± 65.94	0.4736	0.08356
Leptin (pg/ml)	154.10 ± 18.52	Adalimumab	428.61 ± 119.19	430.58 ± 125.22	386.39 ± 89.73	361.91 ± 87.36	339.04 ± 77.16	0.0392*	0.7467
		Etanercept	523.24 ± 433.79	458.87 ± 3325.35	446.79 ± 301.40	422.12 ± 298.61	408.61 ± 312.81	0.0010*	0.00110*
		Infliximab	755.27 ± 576.68	536.35 ± 502.47	441.66 ± 265.91	358.48 ± 225.14	361.19 ± 235.69	0.0003*	0.00234*
Resistin (ng/ml)	3.05 ± 0.28	Adalimumab	4.48 ± 0.51	3.36 ± 0.36	3.17 ± 0.42	3.35 ± 0.36	2.94 ± 0.31	0.0289*	0.0033*
		Etanercept	4.53 ± 2.86	3.64 ± 2.57	3.48 ± 1.55	3.10 ± 1.55	3.03 ± 1.50	0.626	0.0121*
		Infliximab	3.39 ± 1.12	2.56 ± o.73	3.02 ± 2.42	2.15 ± 0.46	2.16 ± 0.75	0.7492	0.0106*

**Figure 1:**
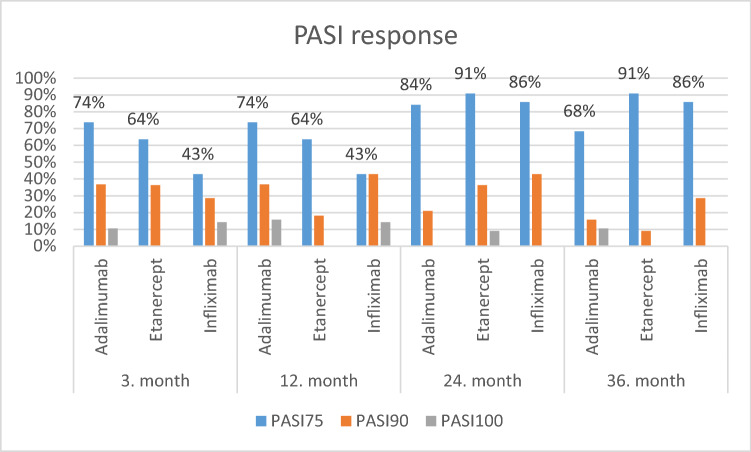
PASI response rates during biologic treatment.

A 75% improvement in PASI (PASI75) was observed at month 3 in 75% of patients treated with adalimumab, 64% treated with etanercept, 43% of patients treated with infliximab. At 36 months of therapy, the highest percentage of improvement in PASI75 was recorded in the group of patients treated with etanercept and infliximab (Table 1, Fig. 1).

**Figure 2:**
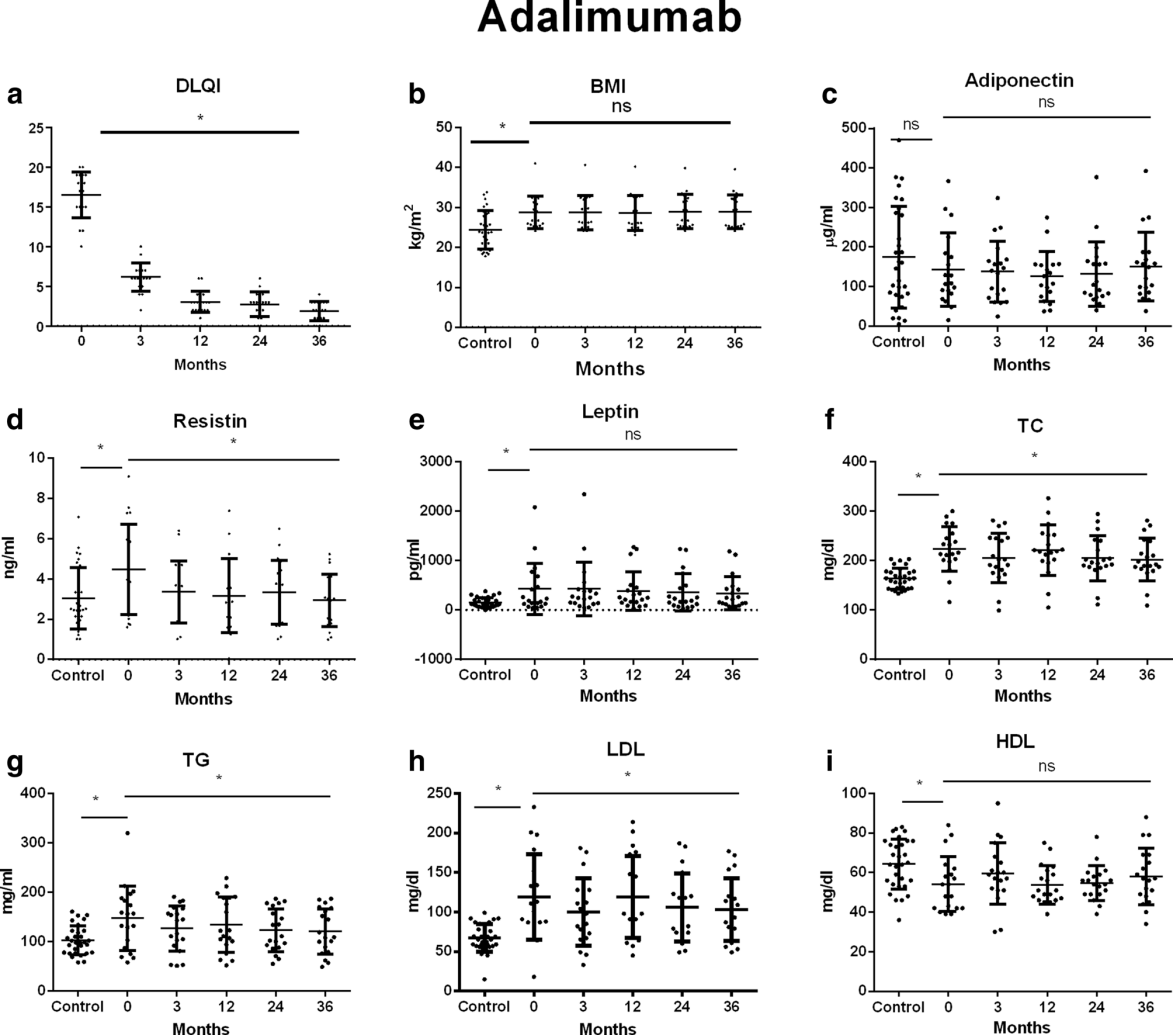
Changes in (**a**) DLQI, (**b**) BMI, (**c**) adiponectin, (**d**) resistin, (**e**) leptin, (**f**) TC, (**g**) TG, (**h**) LDL, (**i**) HDL during treatment with adalimumab. Data showed as means ± SD; DLQI, indicates Dermatology Life Quality Index; BMI, indicates body mass index; TC, indicates total cholesterol; TG, indicates triglyceride; LDL, indicates low-density lipoprotein; HDL, indicates high-density lipoprotein; *p < .05.

**Figure 3:**
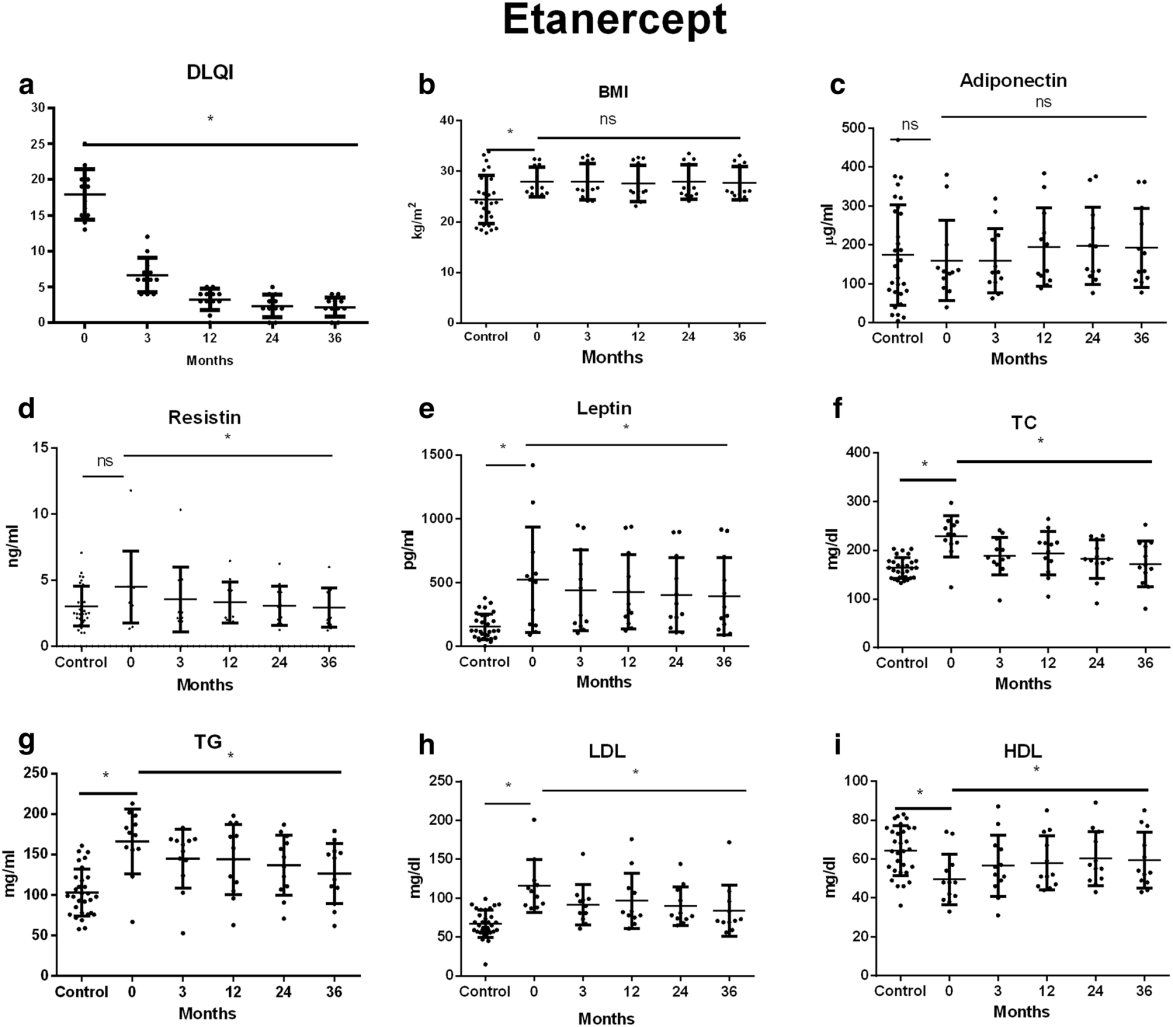
Changes in (**a**) DLQI, (**b**) BMI, (**c**) adiponectin, (**d**) resistin, (**e**) leptin, (**f**) TC, (**g**) TG, (**h**) LDL, (**i**) HDL during treatment with etanercept. Data showed as means ± SD; DLQI, indicates Dermatology Life Quality Index; BMI, indicates body mass index; TC, indicates total cholesterol; TG, indicates triglyceride; LDL, indicates low-density lipoprotein; HDL, indicates high-density lipoprotein; *p < .05.

**Figure 4:**
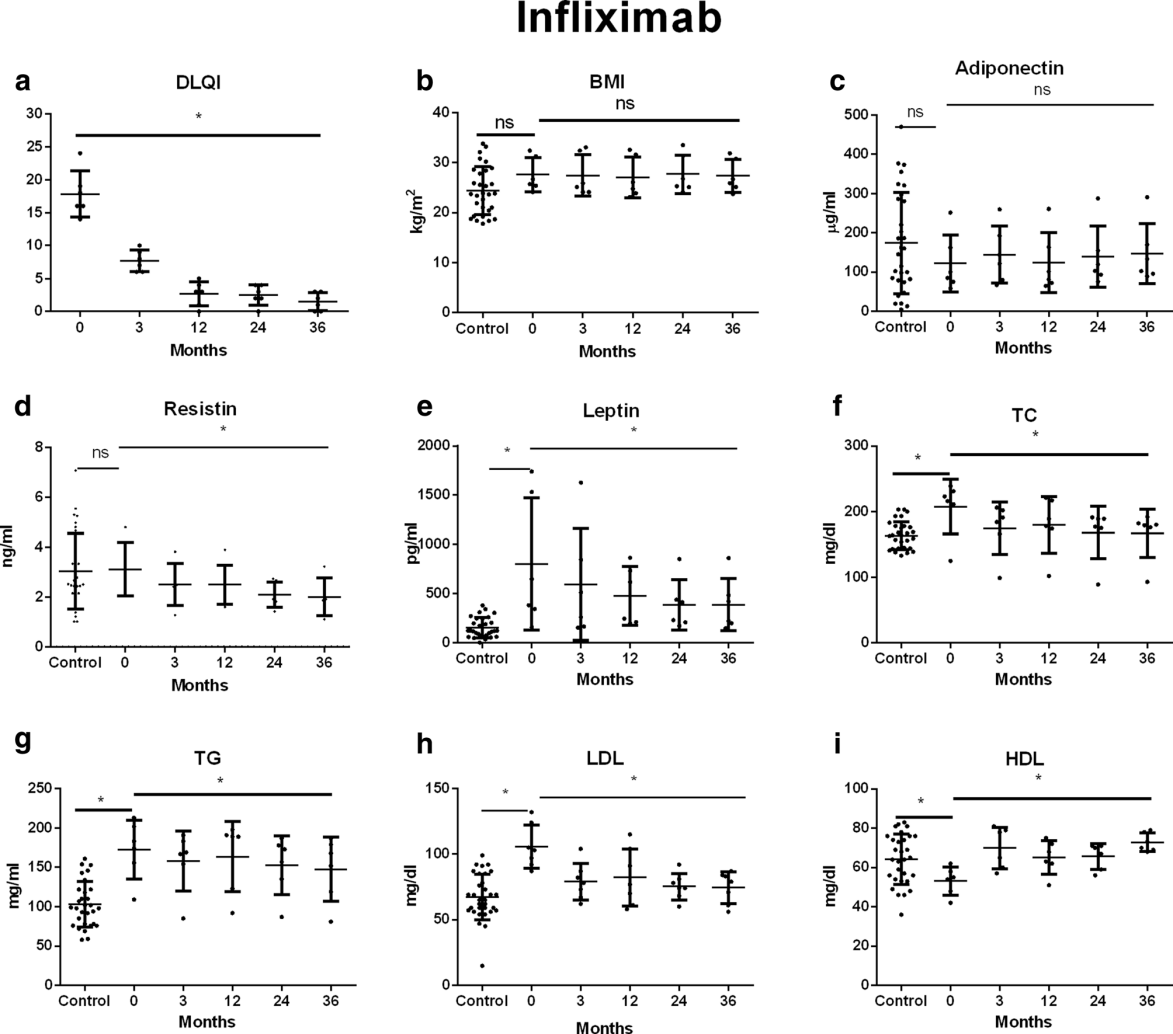
Changes in (**a**) DLQI, (**b**) BMI, (**c**) adiponectin, (**d**) resistin, (**e**) leptin, (**f**) TC, (**g**) TG, (**h**) LDL, (**i**) HDL during treatment with infliximab. Data showed as means ± SD; DLQI, indicates Dermatology Life Quality Index; BMI, indicates body mass index; TC, indicates total cholesterol; TG, indicates triglyceride; LDL, indicates low-density lipoprotein; HDL, indicates high-density lipoprotein; *p < .05.

The quality of life of patients with psoriasis at baseline was significantly reduced. The DLQI value improved significantly after 3 months of treatment with all tested drugs (*p* < 0.05) and remained low until the end of the observation (Table 1, Figs. 2, 3, 4).

The mean body mass index (BMI) of patients with psoriasis before the start of biological therapy in all examined groups was higher than in the control group. There was no significant change in BMI during biological therapy in any of the examined groups (Table 1, Figs. 2, 3, 4).

The mean serum concentrations of adiponectin in patients before treatment were lower than in the control group, however these differences did not reach statistical significance (*p* > 0.05). Therapy with any of the studied drugs did not significantly affect the adiponectin serum concentration (*p* > 0.05) (Table 1, Figs. 2, 3, 4).

Mean serum resistin level in patients with psoriasis prior to treatment with adalimumab was significantly higher than in the control group (*p* = 0.0289). In the remaining groups of patients, concentrations of this adipokine were similar to those observed in the subjects without psoriasis (*p* > 0.05). A significant decrease in serum concentration of this resistin was observed in patients treated with adalimumab, etanercept and infliximab (*p* = 0.003; *p* = 0.001, *p* = 0.01, respectively) (Table 1, Figs. 2, 3, 4).

Prior to the treatment patients presented a significantly higher leptin concentration than the control group. Despite the decrease in mean serum leptin concentrations during biological therapy with all the drugs studied, only the differences observed in the etanercept and infliximab groups reached the statistical significance. (*p* = 0.0011, *p* = 0.00234 respectively) (Table 1, Figs. 2, 3, 4).

The mean total cholesterol in the serum of patients before biological therapy was significantly higher than in the control group (*p* < 0.05). A significant effect of TNF-α inhibitors on total serum cholesterol was noted (*p* < 0.05) (Table 1, Figs. 2, 3, 4).

All observed patient groups showed significantly higher serum triglyceride and LDL levels before the start of therapy than the control group (*p* < 0.05) (Table 1, Figs. 2, 3, 4). They significantly decreased under the influence of biological therapy until the end of the observation (*p* < 0.05) (Table 1, Figs. 2, 3, 4).

Patients in the study group showed significantly reduced HDL levels before treatment when compared to the control group (*p* < 0.05). Among the studied drugs, only etanercept and infliximab significantly influenced the increase of HDL concentration in patients' serum (*p* = 0.00226, *p* = 0.00174) (Table 1, Figs. 2, 3, 4).

We did not observe significant correlation between resistin, adiponectine and leptin serum levels and PASI score, DLQI nor BMI (data not showed).

## Discussion

Systemic inflammation co-existent with skin lesions in psoriasis increases the risk of atherosclerosis and subsequently cardiovascular disease development^10–13^. Under pro-inflammatory conditions, insulin resistance can occur, which also predisposes to atherosclerosis and promotes the formation of adipokines and pro-inflammatory cytokines by the adipose tissue^11,14–16^. Adipokine concentrations observed in patients with psoriasis are similar to those seen in prediabetic subjects^11,16^. These molecules play essential role in systemic inflammation accompanying psoriasis—promoting it or acting in the opposite way. There have been numerous studies investigating the influence of psoriatic treatment on the adipokine levels, but their results are inconsistent^5,10,17–19^.

Adiponectin, is due to recent studies, not only an insulin sensitivity mediator, but also a molecule involved in immune responses. This adipokine regulates dermal inflammation by inhibiting the production of IL-17 (Interleukin 17) by CD4 or CD8-positive T cells^20^. Its levels in the skin as well as in subcutaneous tissue are reduced in patients with psoriasis^20^. According to literature data adiponectin serum concentration in patients with psoriasis is decreased when compared to the general population^5,10^. Previous studies on the effect of psoriasis therapy on adiponectin serum levels have provided conflicting results^5,10,18^. Short-term therapy with methotrexate did not significantly change adiponectin serum concentration in patients, which is consistent with our observations^10^. Campanati et al.^5^ investigated serum levels of various adipocytokines in patients with psoriasis under the therapy with TNF-α inhibitors. After 24 weeks of treatment, only leptin among proinflammatory adipocytokines showed a statistically significant reduction. At the same time adiponectin concentrations were slightly increased but remained significantly lower than in control group. The influence of TNF-α blockade on adiponectine serum concentration in psoriatic arthritis patients was investigated by Peters et al.^18^. Authors did not notice significant change in adiponectine serum level under 12-week therapy with onercept—an antibody against human soluble p55 TNF receptor, though the significant improvement in disease severity. Our study group also presented slightly higher adiponectin serum concentration than the control group, however the difference was not statistically significant. No significant effect of biological therapy on adiponectin serum levels was observed, which is consistent with previous reports.

Resistin is one of the adipokines with pro-inflammatory properties which has the ability to stimulate production of TNF-α and IL-12 by activated B cells. These cytokines along with IL-1β (Interleukin 1β), IL-6 (Interleukin 6) and lipopolysaccharide induce resistin expression by monocytes and macrophages in adipose tissue^7,15^. Higher levels of resistin have been observed in psoriasis patient and its serum concentration was correlated with the severity of skin lesions^17,20,21^. Therefore, it was considered that resistin might be a potential biomarker for diagnosis and prognosis in psoriasis patients^17^. There have been numerous studies on the impact of classical form of psoriatic therapy on the serum resistin level. Kawashima et al.^21^ observed no significant change in leptin concentration, but remarkable decrease in resistin serum level under phototherapy (bath-PUVA—bath-Psoralen Ultra-Violet A and NB–UVB—Narrowband Ultra-Violet B). Although the authors assessed changes in resistin concentrations under the influence of phototherapy, their results are consistent with our observations of the effect of biological therapy on the level of adipokine. Takahashi et al.^22^ investigated the effect of local and systemic psoriasis therapies (topical steroid, topical vitamin D3, narrow band ultraviolet B irradiation, etretinate and ciclosporin) on resistin plasma levels. Resistin concentration decrease following the treatment and was positively correlated with PASI score (r = 0.56). Investigators, however, did not compare changes in adipokine levels between groups of patients undergoing different types of treatment. In other study Takahashi et al.^23^ studied the impact of biological therapy (infliximab, adalimumab, ustekinumab) and NB-UVB phototherapy on adipokines in psoriasis. Adiponectin levels increased significantly, whereas leptin and resistin levels—decreased after 24 weeks of treatment. The effect of therapy on adipokines was similar regardless of the type of treatment used. Authors concluded that improvement of adiponectin, leptin and resistin serum concentrations are linked to effective psoriasis treatment, and this effect is not specific to any type of the therapy. As opposed to the previously mentioned publications, in the study of Pina et al.^19^, 6 months of adalimumab therapy did not significantly change the level of leptin and resistin in the serum of patients with psoriasis. In our study resistin serum level in psoriasis patients was significantly higher than in control group and declined during biologic therapy.

Leptin, one of the major cytokines produced by adipose tissue, is involved not only in body weight regulation but also in immune response^24^. It can induce T-lymphocytes proliferation and stimulate the production of proinflammatory cytokines, such as IL-6 and -TNF-α^25^. Leptin as well as resistin, is involved in Foxp3 + regulatory T-cell (Treg) deficiency in psoriasis, which lead to chronic activation of effector and memory T cells^26–28^. Literature data indicate that serum leptin concentration is positively correlated with body mass index and mass of adipose tissue^11,29,30^. Higher serum concentrations of this adipokine have been shown in patients with psoriasis in comparison with the general population^31^. According to Pina et al.^19^ leptin level is correlated with metabolic syndrome features, while resistin concentration reflects psoriasis severity. Recently published double-blind, randomized trial was conducted to assess the effects of adalimumab and phototherapy in comparison to placebo on the cardiovascular biomarkers for 12 weeks, with subsequent open-label adalimumab extension up to 52 weeks^32^. No significant changes in leptin nor adiponectin serum concentrations during biological therapy was observed. However, patients receiving adalimumab, inversely to our study group, presented a decrease in HDL-P serum levels but no change in total cholesterol and LDL-P. In the present study elevated leptin levels were found in patients with psoriasis which is consistent to previous publications. All investigated drugs contributed to the decrease in leptin levels in patients' sera, but only in the groups treated with etanercept and infliximab these differences reached statistical significance.

Contrary to the previously published studies, average BMI of the observed patients remained stable during the treatment with TNF-α inhibitors. Gisondi et al. reported that psoriatic patients receiving etanercept and infliximab experienced average body mass increase (1.5 ± 2.7 kg and 2.5 ± 3.3 kg respectively)^33^. This effect was not notice in control group receiving methotrexate. The influence of 48-week biologic therapy on patient’s body mass was analyzed by Saraceno et al.^34^. Investigators observed average body mass increase in subjects receiving infliximab (1.54 kg, *p* = 0.0001), adalimumab (2.57 kg, *p* = 0.0014), and etanercept (2.18, *p* = 0.007)^33^. These observations are consistent with those made by Renzo et al. who investigated nutritional status in patients with psoriasis (PsO) and psoriatic arthritis (PsA) treated with etanercept and infliximab^35^. After 24 weeks of anti-TNF-α agents treatment significant weight gain of 2.6 ± 3.2% in the PsO and 2.1 ± 3.5% in PsA group was noted^36^. In the study conducted by Prignano et al. average body mass increase in patients receiving TNF- α blockers (etanercept and infliximab) was reported, however these changes did not reach statistical significance^37^. The lack of the influence of biologic therapy on the average weight of analyzed group of patients may be due to the relatively small number of subject studied.

Numerous reports have demonstrated the dependence of serum adipokines on patients BMI^37–40^. Some works, however, did not show such a correlation^5^. The lack of influence of biological therapy on body mass of the studied subjects could be one of the main factors determining the lack of changes in adiponectin serum concentration in patients.

### Limitations

In this study, we assessed the long-term changes in serum adipokines concentrations in patients under real-live conditions in long-term observation, therefore the study has some limitations. The main one is a relatively small study group which affects the strength of the scientific evidence. Each patient was instructed to follow a healthy diet, but the researchers had no control over the dietary behavior of the study participants. Patients’ dietary habits could influence the obtained results. Next important study limitation is that the control group was not matched by the BMI, which could also distort the obtained results. On the other hand, some previous studies showed that the parameters of lipid metabolism and adipokines are increased in psoriasis, regardless of the body weight of the patients^5^.

## Materials and methods

The study group consisted of 37 patients with moderate-to-severe plaque psoriasis treated with biological therapy in the Department of Dermatology and Venereology, Medical University of Lodz from which blood samples were taken before and in the 3rd, 12th , 24th, and 36th month of therapy. Nineteen patients were treated with adalimumab (5 K, 14 M, age 48.6 ± 13.4 years), 11 with etanercept (5 K, 6 M, age 50.6 ± 11.5), and 7 with infliximab (5 K, 2 M, age 49.9 ± 12.5). All patients included into the study had documented history of inadequate response to at least two classical methods of systemic treatment (cyclosporine, methotrexate, acitretin or PUVA) or had contraindications to them. Washout periods for prior treatments were 4 weeks for systemic therapies and 2 weeks for topical treatment. Thirty age- and gender-matched volunteers without psoriasis were recruited into the control group. Exclusion criteria for both groups were age under 18 years, pregnancy or breast-feeding, and an accompanying immunodeficiency. Neither patients nor controls were taking antilipemic drugs, dietary supplements, and were not following any specific dietary restrictions (food allergies, religious constraints or particular diet regimen). Each study participant was fully informed of the principles of a healthy diet and the dietary recommendations in psoriasis. Characterization of the study group is included in the Table 2.

**Table 2:** Characterization of the study group.

	**Adalimumab**	**Etanercept**	**Infliksymab**	**All patients**	**Control**
n	19 (7 K, 12 M)	11 (5F, 6 M)	7 (5F, 2 M)	37 (17 K, 20 M)	30 (11F, 19 M)
Age (years) (Mean ± SEM)	48.6 ± 13.4	50.6 ± 11.5	49.9 ± 12.5	49.7 ± 12.5	45.4 ± 10.1
Psoriasis duration (years) (Mean ± SEM)	19.1 ± 11.2	18.0 ± 13.2	23.3 ± 13.4	20,1 ± 12.6	–
BMI (kg/m2) (Mean ± SEM)	28.7 ± 0.9	28.0 ± 3.0	28.1 ± 3.2	28.3 ± 2.4	24.41 ± 0.87
Psoriatic arthritis n (%)	6 (31.6)	9 (81.8)	4 (57.1)	19 (51.3)	–
Hypertension n (%)	10 (52.6)	5 (45.5)	4 (57.1)	19 (51.4)	8 (26.6)
Ischemic heart disease n (%)	3 (15.8)	1 (9.1)	1 (14.3)	5 (13.5)	2 (6.7)
History of acute coronary syndrome n (%)	1 (5.2)	0	0	1 (2.7)	0
Type 2 diabetes n (%)	1 (5.2)	1 (9.1)	0	2 (5.4)	1 (3.3)
Glaucoma n (%)	1 (5.2)	1 (9.1)	0	2 (5.4)	0
Cigarette smoking n (%)	7 (36.8)	5 (45.5)	2 (28.6)	14 (37.8)	3 (10)
Obesity n (%)	7 (36.8)	4 (36.4)	3 (42.9)	14 (37.8)	5 (16.7)
Crohn's disease n (%)	0	0	1 (14.3)	1 (2.7)	0
Hyperthyroidism n (%)	0	0	0	1 (2.7)	1 (3.3)
Peripheral artery disease n (%)	1 (5.2)	1 (9.1)	0	2 (5.4)	0

The study was conducted in accordance with the Good Clinical Practice (GCP) guidelines. The experimental plan was conducted according to the Declaration of Helsinki principles. This study was approved by the Bioethics Committee of Medical University of Lodz. Written informed consents were obtained from all the subjects.

Fasting blood samples were taken from all subjects at the beginning of the observation in order to assess lipid profile (TG, total cholesterol, LDL, HDL). The remaining part of the collected blood sample was centrifuged for 15 min at 1000×*g*. The serum samples were then divided into small portions for storage at -80 °C further analysis. Thawed samples were tested in duplicate with the enzyme immunoassay (ELISA) according to the manufacturer's instructions. Serum adiponectin, resistin and leptin concentrations were determined by ELISA technique (QUANTIKINE, R&D Systems, Minneapolis, USA) with a sensitivity of 0.891 ng/ml, 0.055 ng/ml, and 7.8 pg/ml, respectively. Concentrations of leptin in serum were expressed in pg/ml, while adiponectin—in μg/ml, and resistin—in ng/ml.

After a 3-, 12-, 24- and 36-months of the continuous biologic therapy, blood samples were drawn from the patients, and the levels of lipids and adipokines were re-evaluated. At the same timepoints BMI (calculated as the body weight of participants divided by the square of height), psoriasis severity by the PASI Index and quality of life (QoL) by the DLQI Index were assessed.

### Statistical analysis

All analyses were performed using Statistica 13 software (STATSOFT Inc., Tulsa, OK, United States). Intergroup differences for each variable were analyzed using both the Friedman test (nonparametric ANOVA) and repeated measures ANOVA followed by Mauchly sphericity test and, if necessary, Greenhouse–Geisser/Huyhn–Feld correction and multivariate tests (Wilks lambda). Differences between groups were compared using an unpaired Welch’s t-test if the data showed a normal distribution; otherwise the Mann–Whitney U test was used. Pearson’s correlation coefficient was used in correlation analyses between adipocytokines serum levels and PASI score. Data are expressed as mean ± SD, and a *p* value of 0.05 or less was considered as statistically significant.

## Conclusions

Higher leptin and resistin and lower adiponectin concentrations in psoriasis can be a proof of close relationship between skin inflammation and metabolic status in psoriasis patients. Statistically significant decrease in serum resistin proves that biologic therapy not only provide clinical improvement but also affects the systemic inflammation associated with psoriasis and this effect persists with long-term therapy. Proper control of psoriasis provided by TNF-α inhibitors may contribute to decreased risk of cardiovascular diseases in treated patients.

Obtained results are promising, however, further assessment is needed on a larger group of patients.
